# Supplementary material for: A person-centered approach to cognitive performance analysis in primary school children: Comparisons through self-organizing maps
Source: PLoS One. 2025 Feb 20;20(2):e0318836. doi: 10.1371/journal.pone.0318836 (PMC11841877; doi:10.1371/journal.pone.0318836)
Supplement: Attachment — Submitted filename: Response_to_Reviewers_auresp_2.docx [file pone.0318836.s003.docx]

**Journal requirements:**

1. Please review your reference list to ensure that it is complete and correct. If you have cited papers that have been retracted, please include the rationale for doing so in the manuscript text, or remove these references and replace them with relevant current references. Any changes to the reference list should be mentioned in the rebuttal letter that accompanies your revised manuscript. If you need to cite a retracted article, indicate the article’s retracted status in the References list and also include a citation and full reference for the retraction notice.

Author response (AR): All references included in the manuscript have been verified. The following citations have been updated:

- The next reference has been added: 47. Newton M, Duda JL, Yin Z. Examination of the psychometric properties of the perceived motivational climate in sport questionnaire-2 in a sample of female athletes. J Sports Sci. 2000;18: 275–290. doi:10.1080/026404100365018
- The next reference has been added: 56.	Seifert R, Pellicer-Chenoll M, Antón-González L, Pans M, Devís-Devís J, González L-M. Who changed and who maintained their urban bike-sharing mobility after the COVID-19 outbreak? A within-subjects study. Cities. 2023;137: 104343. doi:10.1016/j.cities.2023.104343

**Review Comments to the Author**

5. Is the manuscript presented in an intelligible fashion and written in standard English?

PLOS ONE does not copyedit accepted manuscripts, so the language in submitted articles must be clear, correct, and unambiguous. Any typographical or grammatical errors should be corrected at revision, so please note any specific errors here.

Reviewer #2: No

AR: Thank you for your observation. Although no specific errors were mentioned, we conducted a thorough review of the manuscript, focusing on clarity, grammatical accuracy, and adherence to academic English standards. As part of this process, we carefully identified and corrected any grammatical or typographical errors to ensure the text is clear and unambiguous. Additionally, we refined sentence structure, improved terminology, and addressed potential redundancies to enhance overall readability. We believe these revisions have significantly improved the manuscript and hope they adequately address your concerns.

Reviewer #2:

1. In terms of references, please check the journal guidelines, authors have names and numbers in the text, so please correct it according to journal standards;

AR: We appreciate your observation regarding the referencing style in our manuscript. We have carefully reviewed both the references in the text and the journal's guidelines to ensure compliance. According to the journal's instructions, the Vancouver citation style is used, which allows for numbered citations in square brackets. The guidelines explicitly state the following regarding in-text citations: "In the text, cite the reference number in square brackets (e.g., ‘We used the techniques developed by our colleagues [19] to analyze the data’)."

Based on this information and the regulations stipulated by the Vancouver citation style, we confirm that the citation format we employed—combining author names and citation numbers within the text—is correct (e.g., “O’Malley et al. [31] identified…”). We have thoroughly verified that our manuscript adheres to this format throughout.

If there are specific instances where the referencing style seems unclear or inconsistent, we would greatly appreciate it if you could point them out so that we can address them. Otherwise, we believe the current referencing format aligns with the journal's requirements.

2. In line 168, please describe which specific measurements were performed;

AR: In order to specify the measurements that were carried out during the aforementioned week of measurements, the subsequent sentence in which the measurements were directly mentioned without reference to the specific time of the measurements has been modified. Consequently, the following sentence has been revised and incorporated to clarify this aspect:

Line 164-166: “During this week, data were collected on participants' daily PA levels using accelerometers, alongside written questionnaires, physical tests, and cognitive tests.”

3. Please provide the values of validity and reliability of the questionnaires used for the children;

AR: We appreciate your suggestion. Following your proposal, we have thoroughly revised the manuscript and included the validity and reliability values of the questionnaires. These values have been added to the Table 1 (line 202), where they are mentioned, to ensure that the psychometric properties of each instrument are clearly detailed. We hope that these additions address your concerns and improve the clarity and rigor of the manuscript.

4. Would it be possible for any kind of graphical representation of the Self-Organizing Maps? it’s quite difficult to understand its assessments and how it was applied...

AR: Thank you for your valuable suggestion. We agree that a graphical representation can improve the understanding of the Self-Organizing Maps process and its application. To address this, we have included Figure 1 in the revised manuscript (line 272). This flowchart illustrates the key steps in constructing and training the neural network, as well as the iterative adjustments of the neuronal weights. We believe this addition provides clarity and enhances the accessibility of the methodology for readers.
